# Exploring Metabolic Signature of Protein Energy Wasting in Hemodialysis Patients

**DOI:** 10.3390/metabo10070291

**Published:** 2020-07-16

**Authors:** Fatin Athirah Pauzi, Sharmela Sahathevan, Ban-Hock Khor, Sreelakshmi Sankara Narayanan, Nor Fadhlina Zakaria, Faridah Abas, Tilakavati Karupaiah, Zulfitri Azuan Mat Daud

**Affiliations:** 1 Department of Dietetics, Faculty of Medicine and Health Sciences, Universiti Putra Malaysia, Serdang 43400, Malaysia; fatinpauzi0505@gmail.com; 2 Dietetics Program, School of Healthcare Sciences, Faculty of Health Sciences, Universiti Kebangsaan Malaysia, Kuala Lumpur 50300, Malaysia; sham0901@gmail.com; 3 Department of Medicine, Faculty of Medicine, Universiti Kebangsaan Malaysia, Cheras 56000, Wilayah Persekutuan Kuala Lumpur, Malaysia; khorbanhock@gmail.com; 4 School of BioScience, Taylor’s University, Subang Jaya 47500, Malaysia; sreelakshmiprem07@gmail.com (S.S.N.); tilly_karu@yahoo.co.uk (T.K.); 5 Department of Medicine, Faculty of Medicine and Health Sciences, Universiti Putra Malaysia, Serdang 43400, Malaysia; n_fadhlina@upm.edu.my; 6 Department of Food Science, Faculty of Food Science and Technology, Universiti Putra Malaysia, Serdang 43400, Malaysia; faridah_abas@upm.edu.my

**Keywords:** protein energy wasting, hemodialysis, metabolomics, ^1^H-NMR, metabolic pathways, plasma metabolites

## Abstract

End-stage renal disease patients undergoing maintenance hemodialysis (HD) are vulnerable to the protein energy wasting (PEW) syndrome. Identification and diagnosis of PEW relies on clinical processes of judgment dependent on fulfilling multiple criteria drawn from serum biochemistry, weight status, predictive muscle mass, dietary energy and protein intakes. Therefore, we sought to explore the biomarkers’ signature with plasma metabolites of PEW by using ^1^H-nuclear magnetic resonance for an untargeted metabolomics approach in the HD population, to understand metabolic alteration of PEW. In this case-controlled study, a total of 53 patients undergoing chronic HD were identified having PEW based on established diagnostic criteria and were age- and sex-matched with non-PEW (*n* = 53) HD patients. Fasting predialysis plasma samples were analyzed. Partial least square discriminant analysis demonstrated a significant separation between groups for specific metabolic pattern alterations. Further quantitative analysis showed that the level of 3-hydroxybutyrate, acetate, arabinose, maltose, ribose, sucrose and tartrate were significantly increased whilst creatinine was significantly decreased (all *p* < 0.05) in PEW subjects. Pathway analysis indicated that PEW-related metabolites reflected perturbations in fatty acid mechanism and induction of glyoxylate and dicarboxylate pathway attributed to gluconeogenesis. These results provide preliminary data in understanding metabolic alteration of PEW and corresponding abnormal metabolites that could potentially serve as biomarkers of PEW.

## 1. Introduction

Protein energy wasting (PEW) syndrome is characterized by a persistent depletion of protein stores and energy fuels in the body, caused by multiple nutritional and metabolic alterations [1] arising from the accumulation of uremic solutes. The syndrome is common to patients with advanced chronic kidney disease (CKD) undergoing dialysis treatment [2]. This state of nutritional and metabolic perturbations among dialysis patients progressively degrades their quality of life and reduces the survival [3] attributed to increased risks for cardiovascular pathogenesis, infection, inflammation and hospitalization [4,5,6].

The term PEW as a uniform nomenclature was proposed by the International Society of Renal Nutrition and Metabolism (ISRNM 2008), after increasing research reported on pathophysiological mechanisms leading to the syndromes of muscle wasting, malnutrition and inflammation in CKD patients [3,7]. Accordingly, ISRNM proposed that PEW diagnosis concurs with three out of four proposed categories; low levels of biochemical indicators (i.e., albumin, transthyretin or cholesterol), decreased body mass (i.e., body mass index, unintentional weight loss and body fat percentage), reduced muscle mass (i.e., muscle wasting or sarcopenia and reduced mid-arm muscle circumference) and unintentional low dietary intake (i.e., energy and protein intake) [7]. The unifying concept of these diagnostic domains is an indirect assessment for body protein and energy depletion.

Identification of PEW thus far has relied on clinical judgment processes based on an extensive patient assessment. However, the application of the ISRNM diagnostic criteria to diverse hemodialysis (HD) populations is challenging [3], due to reliance on the indirect measurement of muscle mass using skinfold measurements and norms developed for Caucasian populations [8,9], as well as high prevalence of underreporting of dietary energy and protein intakes [10,11]. It is also noted that ‘serum chemistry’ biomarkers proposed within ISRNM diagnosis criteria are not sufficient or specific enough [12,13,14,15,16] to reflect and detect the PEW syndrome. Basically, the fundamental understanding of PEW in this uremic patient population remains unresolved as studies explaining the mechanistic pathways are scarce. Therefore, to counteract these issues relating to PEW diagnosis, a potential and comprehensive clinical approach that complements the ISRNM recommended criteria needs to be developed and established, particularly in elucidating mechanisms relating to net body protein losses [17].

In line with this need, we propose to use the cutting edge ^1^H-nuclear magnetic resonance (^1^H-NMR)-based metabolomics approach to facilitate identification of plasma metabolites related to the catabolism of muscle and fat tissues associated with facets of PEW development in HD patients. The metabolomics approach would facilitate the identification of a consistent, precise and reliable marker to improve the diagnosis of PEW. This unbiased quantitative method would detect abnormal metabolites related to PEW potential biomarkers, as well as clarify the underlying pathophysiological mechanism. The fact is, the metabolome represents the ultimate downstream products of cellular metabolism [18,19] and thus changes in metabolites should be the closest reflection of the phenotype [20] to elucidate the PEW mechanism [21].

Metabolomics via the NMR platform offers a simple sample preparation for a convenient but comprehensive analysis for the identification of metabolite biomarkers for the diagnosis of PEW. Importantly, no study pertaining to identification of PEW metabolites using the metabolomics approach has been published to date. Therefore, this study sought to identify biomarker signatures of PEW using the ^1^H-NMR based metabolomics approach to understand the metabolic alteration associated with PEW in maintenance HD patients.

## 2. Results

The demographic characteristics, clinical and biochemistry data of the study population are presented in Table 1. Patients are equally distributed between PEW and non-PEW (NPEW) groups based on the age, gender and ethnicity (all *p* > 0.05). On the other hand, the PEW group exhibited a significantly higher trend towards a longer time on dialysis (*p* = 0.041), but lower trends of creatinine (*p* < 0.001) and blood urea (*p* = 0.036) levels compared to the NPEW group. The PEW group also demonstrated heightened inflammatory responses with higher IL-6 values (*p* = 0.006), although high sensitivity C-reactive protein (hsCRP) levels between groups was not significant (*p* > 0.05). Insulinemic status as per plasma insulin levels and homeostasis model assessment index of insulin resistance (HOMA-IR) scores were both significantly lower (*p* < 0.001) for the PEW compared to the NPEW group.

In comparing the two groups based on the ISRNM-PEW criteria, the PEW group had significantly lower values for all nutritional indices (*p* < 0.05) except for total cholesterol (TC) and dietary protein intake (DPI) (Table 2).

### 2.1. Metabolic Profiles Separation between Groups Using Multivariate Data Analysis

^1^H-NMR spectra were obtained for all 106 samples and spectra profiles of plasma samples from hemodialysis patients with PEW and non-PEW (NPEW) are presented in Supplementary Materials Figure S1. An unsupervised principal component analysis (PCA) was performed to determine the possibility of discriminating HD patients presenting with and without PEW. The primary PCA-X score plot (Supplementary Materials Figure S2a) indicated that metabolomics profiles between the two groups were similar as evident by the scattered and random scores from both groups within the same without pattern discrimination. After excluding nine significant outliers from the analysis a new PCA-X model was developed (Supplementary Materials Figure S2b). A similar approach in excluding outliers has also been documented elsewhere [22]. Interestingly, from the overview, the score plot indicated some pattern of scores between the two groups despite perceptible overlaps.

**Figure 1:**
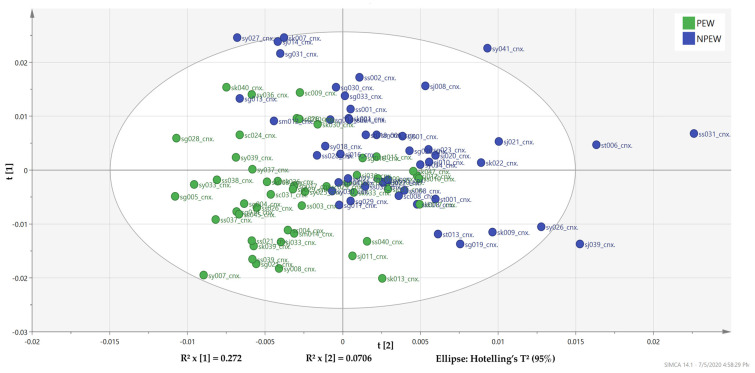
Characterization of the plasma metabolic changes in PEW and non-PEW hemodialysis (HD) patients. Partial least square-discriminant analysis (PLS-DA) score plot indicating metabolomics profile between the two groups (green = PEW; blue = NPEW) with each score representing one subject. The eclipse represents the 95th percentile of confidence interval, while any score outside of the eclipse is considered an outlier. Nine outlier scores were excluded from the analysis. Metabolomics profile of the two groups was clearly discriminated as indicated by a clear separation trend.

Further analysis using supervised partial least square-discriminant analysis (PLS-DA) was performed to maximize the group separation and facilitate the detection of discriminating metabolites in PEW and NPEW patient samples. Figure 1 demonstrated that the PLS-DA score plot clearly showing distinct discrimination in plasma metabolites between PEW and NPEW groups along the principal component 2 (PC2) direction with the goodness of fit R^2^X_(cum)_ = 0.342, R^2^Y_(cum)_ = 0.426 and Q^2^_(cum)_ = 0.222. This model was acceptable as the difference between R^2^Y_(cum)_ and Q^2^_(cum)_ was <0.3, indicating each sample was equally and uniformly contributing to the observed group separation [23]. The application of the PLS-DA model was validated with the use of permutation tests with 100 iterations. The resulting R^2^Y-intercept value that was less than 0.3–0.4 and the Q^2^Y-intercept value of <0.05 supported the PLS-DA model validity, as values larger than 0.05 indicated overfitting [24] in the original PLS-DA model (Supplementary Materials Figure S3a,b). Moreover, cross-validated analysis of variance (CV ANOVA) based on cross-validated predictive residuals indicated the level of significance of group separation in the model (Supplementary Table S1) resulted in a *p* value of <0.05 denoting a statistically significant model of PLS-DA [25]. The PLS-DA score plot provided a stronger clustering for the PEW group on the negative side of PC2, suggesting that the metabolic profiles had changed. Therefore, it can be inferred that the PEW syndrome induced specific metabolic pattern changes resulting in different profiles between groups. In addition to further identifying the metabolites responsible for the differences in metabolic profiles between PEW and non-PEW groups, the corresponding loading plot and variable importance in projection (VIP) statistics with a VIP score >1 were used.

**Figure 2:**
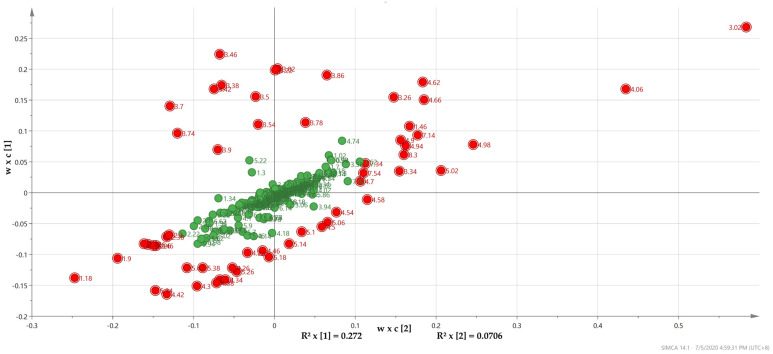
Corresponding loading plot of PLS-DA with a confluence of variable importance in the projection (VIP) results (highlighted as red). The loading plot reveals the important regions in the spectra (metabolites) are responsible for the group clustering shown by the score plot. These regions matched those identified by the VIP plot (VIP > 1) and lead to the separation of the groups.

Figure 2 shows that the most important NMR spectral regions (ppm) driving the observed group separation identified by the VIP plot (VIP > 1) were similar to those delineated by the PLS-DA loading plot, validating the consistency of the data. Hence, potential discriminating markers were acquired from the confluence of corresponding loading and VIP plots from PLS-DA, and markers were selected based on their contribution to the variation.

### 2.2. Metabolites Identification and Quantification

With the completion of the PLS-DA analysis, discriminating metabolites responsible for the differences in the metabolic profiles between the two groups were extracted. By comparing to the database provided in Chenomx NMR Suite software, a total of 32 metabolites causing the distinct separation were identified and quantified, with 18 metabolites belonging to the PEW group and 14 metabolites representing the NPEW group (Supplementary Materials Table S2). After quantification of each metabolite, values were subjected to the Mann–Whitney U analysis. We identified a few metabolites that were significantly increased (*p* < 0.05) in PEW patients as compared to NPEW patients including 3-hydroxybutyrate (*p* < 0.001), acetate (*p* = 0.027), arabinose (*p* = 0.029), maltose (*p* = 0.021), ribose (*p* = 0.041), sucrose (*p* = 0.008) and tartrate (*p* = 0.018). In contrast, creatinine (*p* < 0.001) was the only metabolite that showed a significantly lower plasma concentration in PEW patients compared to NPEW patients (Table 3). Hence, it can be concluded that the group differentiation between the PEW and NPEW groups was highly affected by these metabolites.

### 2.3. Metabolic Pathway Analysis

**Figure 3:**
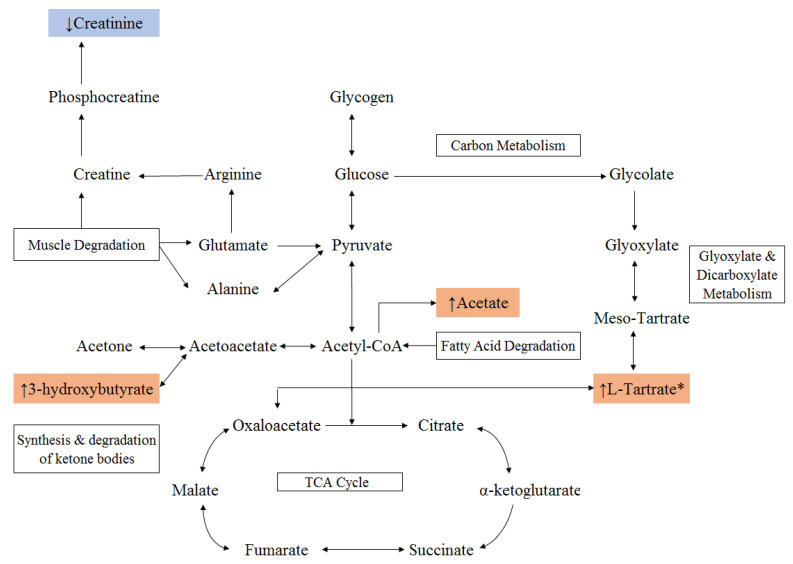
Proposed metabolic pathways of the PEW condition. The figure shows the correlation network of significantly altered metabolites detected in plasma of PEW group patients. The disordered metabolic pathways are fatty acid β-oxidation and gluconeogenesis. The changes of metabolites in PEW subjects are shown in orange (significantly increased) and blue (significantly decreased) as compared to NPEW groups. * Tartrate significance is diminished after adjustment for covariates.

In order to gain insight on the metabolic mechanism of PEW, the Kyoto Encyclopedia of Genes and Genomes (KEGG) database were referenced to identify metabolic network(s) and biological relevance of the significant metabolite changes in PEW patients. The significant pathways identified by KEGG were fatty acid β-oxidation and gluconeogenesis. However, other metabolic pathways such as glyoxylate and dicarboxylate metabolism and carbon metabolism were also implicated (Figure 3). It may be postulated that these target pathways showed either marked perturbations or activation in response to the PEW syndromes in Malaysian HD patients.

### 2.4. Analysis of Covariance (ANCOVA)

Additionally, a one-way analysis of covariance (ANCOVA) was also performed to identify significant discriminating metabolites between the two groups after adjustment for covariates of age and dialysis vintage. Results indicated that specific metabolites 3-hydroxybutyrate, acetate and creatinine were significantly different (*p* < 0.05) between the groups, even after adjustment for these covariates (Supplementary Materials Table S3). Interestingly, arabinose, maltose, ribose, sucrose and tartrate were not significantly different between groups after controlling for the covariates although unadjusted means denoted otherwise. This indicated that these covariates had no contribution on the separation of the groups. Subsequent two-way ANCOVA whilst controlling the age and dialysis vintage indicated there was no statistically significant two-way interaction effect (*p* > 0.05) between the group and gender, implying that the effect of group differentiation was not driven by this factor.

## 3. Discussion

The present study, to the best of our knowledge, is the first to report on key differences in metabolic profiles between PEW and NPEW subjects. To begin with, HD patients in both PEW and NPEW groups had comparable demographics and clinical parameters distribution suggesting minimal confounders. The PEW group exhibited the expected derangement in nutritional outcomes based on the ISRNM diagnostic criteria that include lower serum albumin, BMI, mid-arm muscle circumference (MAMC), mid-arm muscle area (MAMA) and dietary energy intake (DEI). Some other parameters including serum creatinine and urea are also lower when compared to their counterpart suggesting more progressive muscle wasting [26], and in fact are associated with poor survival in several studies [26,27]. The PEW group had relatively longer duration on dialysis treatment (dialysis vintage) while exhibiting a higher inflammatory marker (IL-6), in line with other studies, that reported progressive decline in nutritional parameters following the commencement of dialysis treatment [28] and progressive increase in proinflammatory cytokines [29].

In this study, metabolomic analysis showed a significant separation of the metabolites profile in PLS-DA with a stronger clustering for the PEW group on the negative side of PC2 but on the positive side for the NPEW group. This suggests that the PEW syndrome induced that a specific metabolite pattern alteration resulted in different profiles between the groups. As metabolomics identifies and quantifies various groups of endogenous metabolites [30], metabolic profiling of these two patient groups provides an instant view of altered metabolism present in the PEW group and variables responsible for the group differentiation. Quantitative analysis on discriminating metabolites revealed a statistically significant elevation of 3-hydroxybutyrate, acetate, arabinose, maltose, ribose, sucrose and tartrate with a concurrent decrease in creatinine concentration of PEW patients. There is no comparable study that utilized the metabolomics platform to elucidate the PEW mechanism in the HD population, however, prior studies have been able to identify potential early biomarkers of CKD such as trimethylamine N-oxide, kynurenine and citrulline [31,32], indicating abrupt amino acids metabolism in CKD.

Metabolic pathway identification revealed that the PEW condition indicated systemic perturbations of fatty acid metabolism as well as inducing the glyoxylate and dicarboxylate metabolism attributed to gluconeogenesis. This is based on the following postulation: chronic suboptimal intake of the dietary protein and energy as evidenced in PEW patients will promote gluconeogenesis, which mobilizes fatty acids and amino acids from adipose tissue and muscle respectively [33,34]. On top of this, as a direct consequence of the kidneys’ role in regulating the endocrine function, kidney disease per se is implicated in causing abnormalities in the excretion, synthesis and action of many hormones. These include resistance to insulin, growth hormone and insulin-like growth factor, which ultimately lead to a loss of muscle mass in adult CKD patients [1]. In fact, a targeted metabolomic profiling among CKD patients subjected to hyperinsulinemic-euglycemic insulin clamp revealed that CKD was associated with disruption in amino acid metabolism and mitochondrial function that impaired anabolic effects on insulin contributing to PEW and muscle atrophy [35].

It is interesting however to observe that the PEW group in our study had lower insulinemic status (plasma insulin levels and HOMA-IR scores) when compared to the NPEW group. We postulated that this could be due to suboptimal food intake leading to overall reduction in insulin secretion [34]. In order to maintain a continuous source of energy in the patients’ body with hepatic glycogen depletion, gluconeogenesis and fatty acid oxidation becomes increasingly important to supply sufficient essential water-soluble substrates to the brain and other tissues [33,36,37].

In the current study, the level of serum creatinine (SCr) was significantly lower (*p* < 0.001) in PEW patients. About 95% of the creatinine is found in the muscle [38] as a product of muscle metabolism through the breakdown of creatine and phosphocreatine. It appears the loss of muscle mass is the most valid criterion for the presence of PEW [39,40]. Park et al. [26] suggests low SCr level could be considered as a proxy of protein-energy wasting in some clinical situations. Therefore, low creatinine levels in the PEW group may result from the persistent activation of the gluconeogenesis pathway fueled by amino acids released from the muscle tissue degradations [41].

We also found significant elevation of the metabolites β-hydroxybutyrate (*p* < 0.001) and acetate (*p* < 0.05), which are implicated in the fatty acid β-oxidation pathway in patients with PEW. Gluconeogenesis is also fuelled by rapid lipolysis and release of fatty acids from adipose tissue [36]. Fatty acids are converted to CoA thioesters through the β-oxidation process that occurred in the mitochondria. These reactions upregulate acetyl CoA production, but much of the acetyl CoA cannot enter the citric acid cycle as gluconeogenesis reduces the availability of oxaloacetate to generate glucose. As a result, excess acetyl CoA is converted to ketone bodies through the ketogenesis pathway in order to generate energy. With formation of acetoacetyl-CoA from acetyl-CoA, accumulations of both compounds generate HMG-CoA, which cleaves to yield acetoacetate, which is then reduced to β-hydroxybutyrate. Eventually the liver produces ketone bodies copiously as the citric acid cycle fails to oxidize acetyl units generated from fatty acids degradation [36,37]. A significant increase of β-hydroxybutyrate in this study thus suggests PEW triggered perturbations in fatty acid β-oxidation pathway leads to ketogenesis. Excessive ketone bodies formation indicated a switch to severe starvation [1,34,42] leading to hypoalbuminemia and muscle wasting that are apparent in the PEW group. The presence of acid and ketone bodies are paramount in making protein loss from muscle greater than from other organs [1], interfering in normal cellular functioning [43] and inducing oxidative stress [44]. Altogether, this partly explained the accelerated muscle loss, hypoalbuminemia and heightened inflammation present in the PEW group.

Additionally, the accumulation of acetyl-CoA leads to elevated levels of plasma acetate, which is observed in PEW patients. High acetate levels may also indicate a disturbance in the fatty acid β-oxidation pathway, particularly from the action of heart mitochondria. Unlike liver mitochondria, heart mitochondria do not form acetoacetate [45]. Hence, an increase in acetyl-CoA formation from pyruvate leads to the release of acetate especially under conditions where the citric acid cycle is inhibited [46,47]. Acetyl-CoA hydrolase in heart mitochondria are induced to form acetate and thus relieve ‘acetyl pressure’ [48,49]. Circulating acetate serves to redistribute oxidizable substrate similar to ketone bodies. Moreover, another in vivo study has shown that acetate utilization relies on the availability of glucose [50]. Hence, the substantial rise in the concentration of blood acetate may relate to impaired glucose entry into the muscle of the diabetic animal, which eventually limits acetate utilization [50].

An important observation from our study is that the level of tartrate was significantly increased (*p* < 0.05) in PEW patients, although after adjustment with covariates this significance disappeared. Tartrate accumulation in PEW subjects may be ascribed to the endogenous production from either bacterial degradation in the gut or via tissue metabolism or from exogenous substrates such as medicine and food [51,52]. Interestingly, the endogenous increase in tartrate suggests activation of glyoxylate and dicarboxylate metabolism that occur with gluconeogenesis in PEW patients. Butch et al. [53] have shown that meso- and DL-tartrates can be produced from glyoxylate with a reduction of dihydroxyfumarate as a putative intermediate. Our metabolomics analysis also revealed a significant increase (*p* < 0.05) in the arabinose, maltose, ribose and sucrose concentrations in patients with PEW compared to NPEW patients, although after an adjustment with covariates this significance disappeared. The implication of this finding is unknown although it has been shown that longer dialysis vintage is related with unfavorable changes in the nutritional status of HD patients [54], which may alter the generation of metabolites.

In response to suboptimal intakes of dietary protein and energy associated with the PEW condition, the body will promote gluconeogenesis and fatty acid oxidation to supply energy. This would affect the level of specific metabolites involved in the mechanisms. We found significant differences (*p* < 0.05) in the levels of 3-hydroxybutyrate, acetate, tartrate and creatinine to discriminate PEW from NPEW patients, clearly indicating there were aberrations in fatty acid metabolism and evidence for the presence of the gluconeogenesis pathway. Whereas NPEW patients who did not experience a persistent loss of protein and energy stores in their body demonstrated significantly opposite trends (all *p* < 0.05) for these metabolites indicating that utilization of fatty acids and amino acids as the primary energy-yielding fuel was non-essential. As this is a hypothesis generating study with no baseline data on the PEW syndrome, these discriminating metabolites between PEW and NPEW patients could potentially serve as biomarkers of PEW in HD patients.

Altogether, we demonstrated that metabolomics is a powerful tool not only for identifying biomarkers related to the metabolic signature of PEW, but further elucidates the metabolic abnormalities related to PEW. As this is the first study to adopt the metabolomics approach to examine differences in PEW and non-PEW patients on HD, future studies using a targeted approach would be critical in identifying therapeutic approaches that would change the metabolites linked to gluconeogenesis prevalent in PEW patients.

The design of this study is susceptible to selection bias because this is a case-controlled study. Hence, a larger cross-sectional population sampling is required as an important step forward from this current study. Moreover, despite rapid and simple sample preparation for a convenient but comprehensive analysis, NMR spectrometry has a weak point of lower sensitivity [18] compared to mass spectrometry. Hence, study using another metabolomics platform such as the mass spectrometry-based metabolomics technique is required to validate the discriminating metabolites involved in PEW.

## 4. Materials and Methods

This study received ethical approval from the Medical Research Ethical Committee of the Ministry of Health (NMRR-15-865-2560). All patients provided written informed consent.

### 4.1. Subject Selection and Grouping

This was a case-control study based on a subset of data collected for the multicenter Hemodialysis Nutrition Study (HDiNS). The inclusion criteria of the HDiNS study were HD patients above 18 years old, dialyzed for at least 3 months and willing to provide fasting blood samples. Patients with poor adherence to the HD regime, cognitive impairment or terminal illness were excluded. The ISRNM criteria [7] were used for the diagnosis of PEW. Briefly, patients were assessed for low serum levels of albumin, reduced body weight, decreased muscle mass and unintentional low energy and protein intakes and were diagnosed as PEW when three out of four proposed categories are present. A total of 53 HD patients were identified as PEW. A non-PEW patient (control) was individually matched to each PEW patient (case) by age and sex. Therefore, plasma samples for a total of 106 patients were used to statistically discriminate metabolite profiles between PEW (*n* = 53) and non-PEW (*n* = 53) HD patients.

### 4.2. Plasma Samples Collection and Biochemical Analysis

Typically, pre-dialysis blood samples were collected from patients after 8–10 h fasting through their dialysis access site during a mid-week dialysis session. Then plasma was isolated by centrifugation and divided into aliquots. For this case-control study, a total volume of 0.5 mL of plasma aliquot collected in heparin tubes was reserved for metabolomics analysis, as other anti-coagulants such as EDTA and citrate or other added stabilizers give additional signals in the NMR spectra [55]. Samples were snapped frozen with liquid nitrogen before storage at −80 °C until further analysis.

Serum urea and creatinine data was obtained from patients’ medical records. Additionally, the serum high-sensitivity C-reactive protein (hsCRP) was measured by an immunoturbidimetric assay and serum insulin by the electrochemiluminescence assay method by an independent laboratory using automated clinical chemistry (Roche/Hitachi 912 System, Roche Diagnostics, Tokyo, Japan). IL-6 concentrations were measured in-house by the sandwich enzyme-linked immunosorbent assay (ELISA) method using a commercial kit, IL-6 High Sensitivity Human Elisa Kit (Abcam, Cambridge, MA, USA). HOMA-IR was calculated using both serum glucose and serum insulin levels using the following equation [56]:

HOMA-IR = [Serum glucose (mmol/L) × fasting insulin (mU/L)]/22.5,(1)

### 4.3. Sample Preparation for Metabolite Profiling

Upon thawing of frozen samples, a total of 500 μL plasma were vortexed for a minute followed by a centrifugation at 10,000 rpm for 2 min in order to remove solid debris. Plasma supernatant (400 μL) was then transferred into a 0.5 mL 3 kDa centrifugal filter (Merck, Darmstadt, Germany) to remove macromolecules such as proteins, lipids, lipoproteins and the protein-bound form of molecules [57] and spun for 30 min at room temperature for 13,800 rpm. The filters were initially prewashed three times with deionized water to remove glycerol preservative [58]. The filters were inverted and centrifuged at 13,800 rpm for 5 min to remove any remaining water. The ‘filtrates’ were subsequently transferred into fresh 1.5 mL Eppendorf tubes and diluted with phosphate buffer (KH_2_PO_4_) prepared in deuterium oxide (D_2_O) containing 0.2% trimethylsilyl propanoic acid (TSP, Merck, Darmstadt, Germany) and 0.1% imidazole (Sigma-Aldrich, St. Louis, MO, USA) in 1:2 ratio (200 μL filtrate + 400 μL D_2_O). A total of 600 μL of prepared samples were then transferred to 5 mm NMR tubes.

### 4.4. ^1^H-NMR Analysis

NMR analysis method was adapted and optimized from Maulidiani et al. [59]. Briefly, one dimensional ^1^H-NMR spectra were acquired at 499.887 MHz on a 500 MHz Varian INOVA NMR (Varian Inc., Palo Alto, CA, USA) at a temperature of 26 °C. Water signals and broad protein resonances were suppressed by the combination of PRESAT and the Carr–Purcell–Meiboom–Gill (CPMG) pulse sequence. NMR spectra of 10 ppm spectral width were obtained from a total of 128 scans and an acquisition time of 536 s. NMR spectra were then processed with Chenomx NMR suite version 8.3 software (Chenomx Inc. Edmonton, AB, Canada) by applying a consistent setting for all sample spectra; 0.50 Hz of line broadening, autophasing, baseline-corrected (Whittaker spline), referenced to TSP as an internal standard and referenced to imidazole as a pH indicator. Intelligent binning was used to divide the spectral region from 0.50 to 10.00 ppm into equal bins (0.04 ppm). The peak ppm values were calibrated with reference to the TSP signal at 0 ppm. Prior to the analysis, the region of spectra associated with residual water (4.77–4.86 ppm) and imidazole (7.29–7.33 ppm and 8.24–8.32 ppm) were removed. Subsequently, the respective spectra data were converted into a table of common integrals containing a non-negative value for multivariate data analysis.

### 4.5. Multivariate Data Analysis

Multivariate data analysis of the processed spectra was performed using SIMCA-P software version 14.1 (MKS Umetrics, Umea, Sweden). Firstly, data were mean-centered, and Pareto scaled by dividing each variable by the square root of its standard deviation. Principal component analysis (PCA) and partial least square-discriminant analysis (PLS-DA) were used to analyze the spectra data. PCA is an unsupervised statistical analysis used to display the dominating trends of clustering inherent in the data set as well as the outliers [23]. A regression extension of PCA, a PLS analysis, with a focus on maximum separation (PLS-DA) instead of maximum variation was also used [24,25]. Supervised analysis statistics PLS-DA maximized the class discrimination based on X and Y variables with the group identity defined. Hotelling’s T2, which represent multivariate generalization of the 95% confidence interval, was used to identify the outliers defined as any data point falling outside of the eclipse [24]. To test for validity, a permutation test (100 cycle) and ANOVA of cross-validated residuals (CVANOVA) [60,61] were conducted with the PLS-DA model. Loading plots combined with variable importance in the projection (VIP) values of >1 [60] were used to determine the potential biomarkers in PLS-DA.

### 4.6. Metabolites Identification, Quantification and Pathway Analysis

Chenomx NMR suite version 8.3 software (Chemomx Inc, Edmonton, AB, Canada) was used to identify and quantify metabolites. Based on multivariate data analysis, parts of the spectra (ppm) responsible for the difference between groups were identified using Chenomx software by matching the corresponding peak based on the position, intensity and line-width with the 500 MHz metabolites library available from the software. The area under the peak corresponded to the relative concentration of the metabolites. Once the changes in metabolite concentrations had been determined, the metabolic pathway identification was then performed based on the Kyoto Encyclopedia of Genes and Genomes database [62].

### 4.7. Statistical Analyses

Normality of the data was checked prior to data analysis. Data that was not normally distributed was log transformed. The SPSS statistical software (IBM, Chicago, IL, USA) was used for statistical analysis with a *p* value of <0.05 considered to be significant. Data for categorical variables were reported as number and percentage values, while continuous data were reported as mean ± SD. An independent *t*-test was performed to analyze continuous data whilst Chi-square (χ^2^) statistics was used to evaluate differences between categorical variables. A one-way ANCOVA with the Bonferroni post-hoc test was performed to determine any significance between the groups for the identified metabolites when adjusted for covariates.

## 5. Conclusions

In summary, the metabolomics analysis revealed that the plasma metabolic profiles of PEW subjects were altered in response to the syndrome that distinguished PEW from the NPEW group. Significant differences in the levels of 3-hydroxybutyrate, acetate, tartrate and creatinine were clearly ascribed to the presence of PEW, indicating there were aberrations in fatty acid metabolism and the presence of the gluconeogenesis pathway. Overall, these metabolites can be considered as the potential biomarker signature of PEW in HD patients. Although the results need further validation, this study demonstrated the metabolomics approach has the potential to develop a diagnostic tool for PEW syndrome.

## Figures and Tables

**Table 1:** Demographic, clinical and biochemical characteristics of the study population.

Characteristics	PEW Subjects (*n* = 53)	NPEW Subjects (*n* = 53)	*p*-Value
**Demographics**			
Age (years)	55 ± 14	56 ± 14	ns
Gender (males, %)	29 (54.7)	29 (54.7)	ns
Ethnicity			ns
Malay (*n*, %)	8 (15.1)	11 (20.8)	
Chinese (*n*, %)	36 (67.9)	32 (60.4)	
Indian (*n*, %)	9 (17)	10 (18.9)	
**Clinical and Biochemistry**			
Dialysis vintage (months)	89.96 ± 85.52	58.74 ± 56.30	0.041
Kt/V	1.28 ± 0.97	1.17 ± 0.69	ns
Serum creatinine (μmol/L)	712 ± 160	891 ± 214	<0.001
Serum urea (mmol/L)	18.88 ± 4.43	20.88 ± 5.14	0.036
Insulin (mU/L)	6.75 ± 5.29	13.83 ± 5.85	<0.001
HOMA-IR	1.84 ± 1.80	4.23 ± 2.82	<0.001
hsCRP (mg/L)	3.71 ± 3.28	5.73 ± 5.87	ns
IL-6 (pg/mL)	7.15 ± 5.66	4.27 ± 3.58	0.006

Abbreviations: PEW—Protein Energy Wasting group; NPEW—Non-PEW group, Kt/V—index of dialysis adequacy; HOMA-IR—homeostasis model assessment index of insulin resistance, hsCRP—high sensitivity C-reactive protein, IL-6—interleukin 6, ns—not significant. Notes: Patients were stratified into PEW and NPEW based on the International Society of Renal Nutrition and Metabolism (ISRNM) criteria [7]. Data are reported as mean ± SD for continuous variables and number (percentage) for categorical variables. Differences in age, dialysis vintage, Kt/V, serum creatinine, serum urea, insulin, HOMA-IR, hsCRP and IL-6 were tested using an independent *t*-test while differences in gender and ethnicity were tested using a chi-squared test. Statistically *p* < 0.05 is considered significant.

**Table 2:** Characteristics of patients based on ISRNM PEW criteria.

Parameters	Cutoff for PEW Criterion	PEW Subjects (*n* = 53)	NPEW Subjects (*n* = 53)	*p*-Value
**Biochemical**				
Serum albumin (g/L)	<3.8 g/dl	36.45 ± 4.85	39.77 ± 3.62	<0.001
TC (mmol/L)	<2.59 mmol/L	4.21 ± 1.16	4.07 ± 0.93	ns
**Body Mass**				
BMI (kg/m^2^)	<23 kg/m^2^	20.34 ± 2.68	26.76 ± 3.74	<0.001
**Muscle Mass**				
MAMC (cm)	Decreased mid-arm muscle circumference area (reduction >10% in relation to 50th percentile of reference population)	20.31 ± 2.63	24.63 ± 2.68	<0.001
MAMA (cm^2^)	Muscle mass loss >5% over 3 months or >10% over 6 months	24.95 ± 7.68	40.48 ± 10.75	<0.001
**Dietary Intake**				
DEI (kcal/kg IBW/day)	<0.80 g/kg/day for at least 2 months	22.63 ± 4.49	24.67 ± 7.55	0.040
DPI (g/kg IBW/day)	<25 kcal/kg/day for at least 2 months	0.90 ± 0.29	0.94 ± 0.37	ns

Abbreviations: PEW—Protein Energy Wasting group; NPEW—Non-PEW group, BMI—body mass index, MAMA—mid-arm muscle area, MAMC—mid-arm muscle circumference, LTM—lean tissue mass, TC—total cholesterol, DEI—dietary energy intake, DPI—dietary protein intake, ns—not significant. Notes: Data are reported as mean ± SD and differences in serum albumin, TC, BMI, MAMC, MAMA, DEI and DPI were tested using an independent *t*-test. Statistically *p* < 0.05 is considered significant.

**Table 3:** Comparison on the relative concentrations of discriminant metabolites between PEW and NPEW identified by ^1^H NMR.

Metabolites	PEW	NPEW	Differences	*p*-Value	Adjusted *p*^a^
3-Hydroxybutyrate	0.051 ± 0.008	0.024 ± 0.002	0.027	<0.001	0.002
Acetate	0.188 ± 0.006	0.172 ± 0.005	0.017	0.027	0.039
Arabinose	0.228 ± 0.020	0.222 ± 0.034	0.006	0.029	ns
Maltose	0.174 ± 0.014	0.157 ± 0.022	0.0164	0.021	ns
Ribose	0.523 ± 0.035	0.459 ± 0.038	0.0645	0.041	ns
Sucrose	0.144 ± 0.117	0.111 ± 0.010	0.0333	0.008	ns
Tartrate	0.202 ± 0.027	0.182 ± 0.032	0.0202	0.018	ns
Creatinine	0.270 ± 0.008	0.331 ± 0.010	−0.0609	<0.001	<0.001

Abbreviations: ns—not significant. Notes: Values are expressed as mean ± SD and *p* < 0.05 is considered significant. *p*-values are derived from the Mann–Whitney U analysis, to compare mean ranks of metabolites between PEW and NPEW groups. *p*^a^ values are adjusted for age and dialysis vintage using a one-way covariance analysis (ANCOVA).

## Supplementary Materials

The following are available online at https://www.mdpi.com/2218-1989/10/7/291/s1, Figure S1: Representative of ^1^H-NMR CPMG spectra of plasma samples obtained from PEW subjects (a) and NPEW subjects (b), Figure S2: (a) PCA-X score plot for NMR spectra acquired from plasma samples of PEW and non-PEW HD patients, (b) PCA-X score plot with 9 outliers removed for NMR spectra acquired from plasma samples of PEW and non-PEW HD patients, Figure S3: Permutation test for validation of PLS-DA model for (a) PEW groups R^2^Y (0.0, 0.25), Q^2^Y (0.0, −0.11) and (b) NPEW groups R^2^Y (0.0, 0.24), Q^2^Y (0.0, −0.12), Table S1: ANOVA for cross validated residuals (CV ANOVA) of PLS-DA model discriminating PEW and NPEW groups, Table S2: Comparison on the mean concentration of discriminating metabolites identified from plasma samples of PEW and non-PEW HD patients, Table S3: Analysis of Covariance (ANCOVA) between selected independent variables on significant metabolites with controlled covariates.
