# Supplementary material for: Exploring Metabolic Signature of Protein Energy Wasting in Hemodialysis Patients
Source: Metabolites. 2020 Jul 16;10(7):291. doi: 10.3390/metabo10070291 (PMC7408592; doi:10.3390/metabo10070291)
Supplement: Supplementary file 1 [file metabolites-10-00291-s001.pdf]

Supplementary Material

A. **PEW samples**

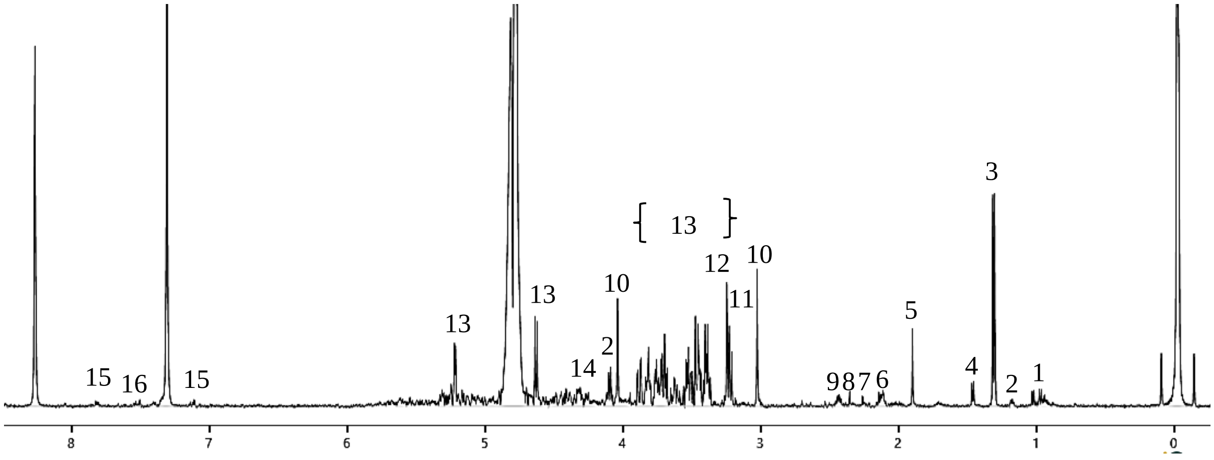

B. **NPEW samples**

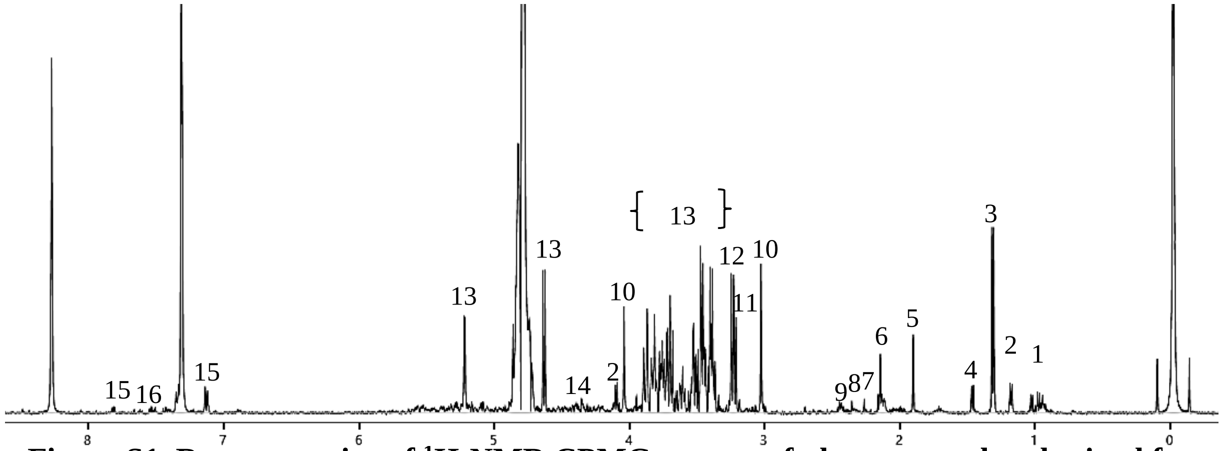

Figure S1. Representative of ^{1}H-NMR CPMG spectra of plasma samples obtained from PEW subjects (a) and NPEW subjects (b)

Note: Metabolites were identified using the Chenomx NMR Suite 8.3 software. Major metabolites identified include 1. Valine, 2. 3-hydroxybutyrate, 3. Lactate, 4. Alanine, 5. Acetate, 6. Hydroxyacetone, 7. Acetoacetate, 8. Pyruvate, 9. 3-Hydroxy-3-methylglutarate, 10. Creatinine, 11. Carnitine, 12. Trimethylamine N-oxide, 13. Glucose, 14. Tartrate, 15. π-Methylhistidine, 16. Mandelate

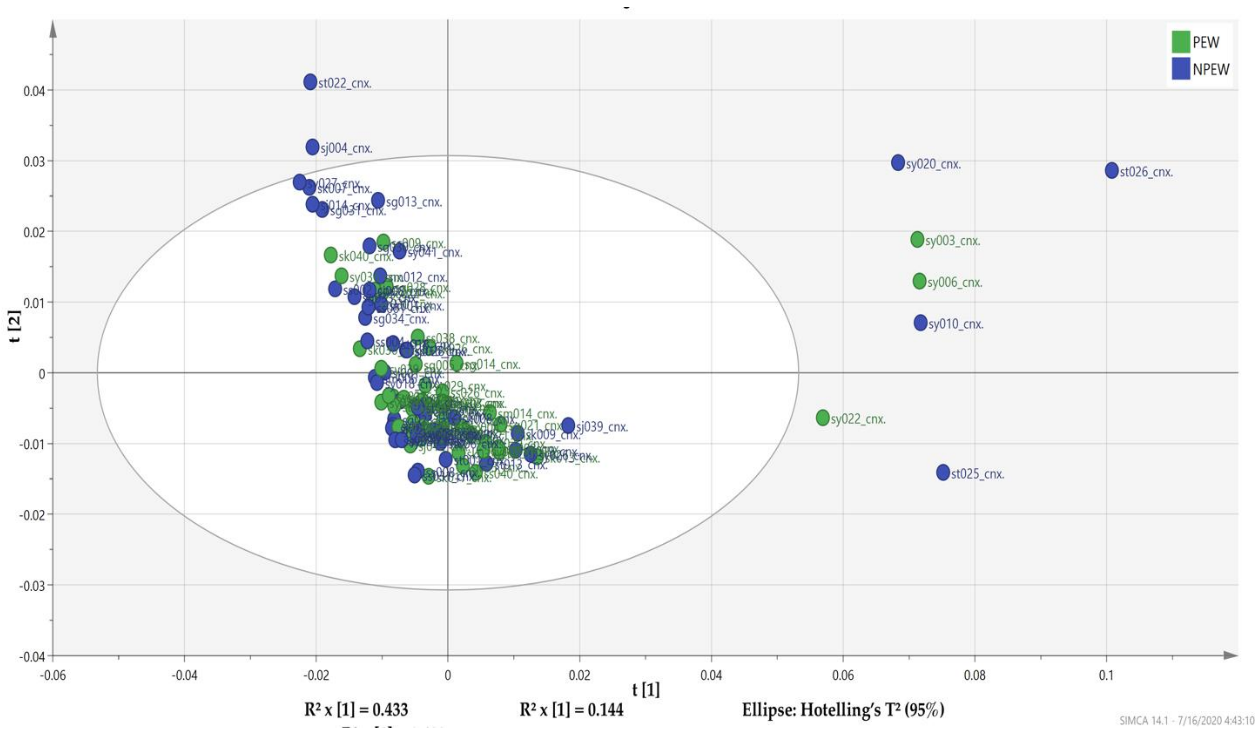

Figure S2a. PCA-X score plot for NMR spectra acquired from plasma samples of PEW and non-PEW HD patients

Abbreviations: PCA: Principal component analysis, PEW: Protein energy wasting, NPEW: Non protein energy wasting

Note: This figure depicts PCA-X score plot indicating metabolomics profile between the two groups with each score representing one subject. The eclipse represents the 95th percentile of confidence interval, while any score outside of the eclipse is considered as an outlier.

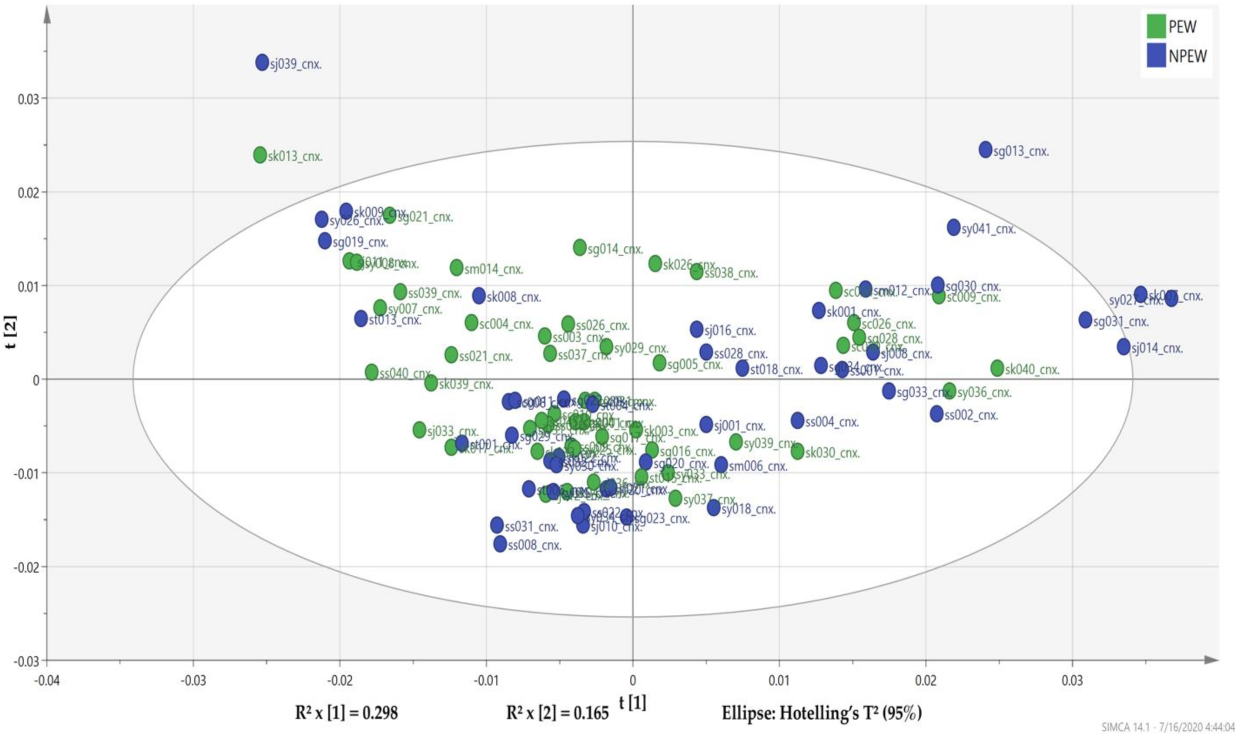

Figure S2b. PCA-X score plot with 9 outliers removed for NMR spectra acquired from plasma samples of PEW and non-PEW HD patients

Abbreviations: PCA: Principal component analysis, PEW: Protein energy wasting, NPEW: Non protein energy wasting

Note: This figure depicts PCA-X score plot indicating metabolomics profile between the two groups with each score representing one subject. The eclipse represents the 95th percentile of confidence interval, while any score outside of the eclipse is consider as an outlier. Nine outlier score was excluded from the analysis.

A) PEW groups

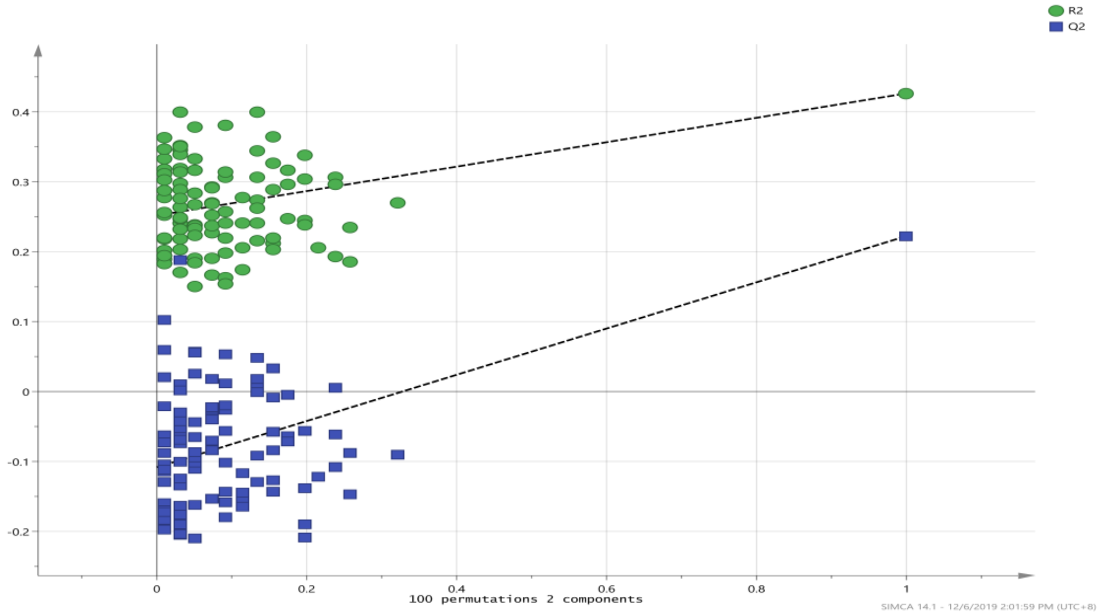

B) NPEW groups

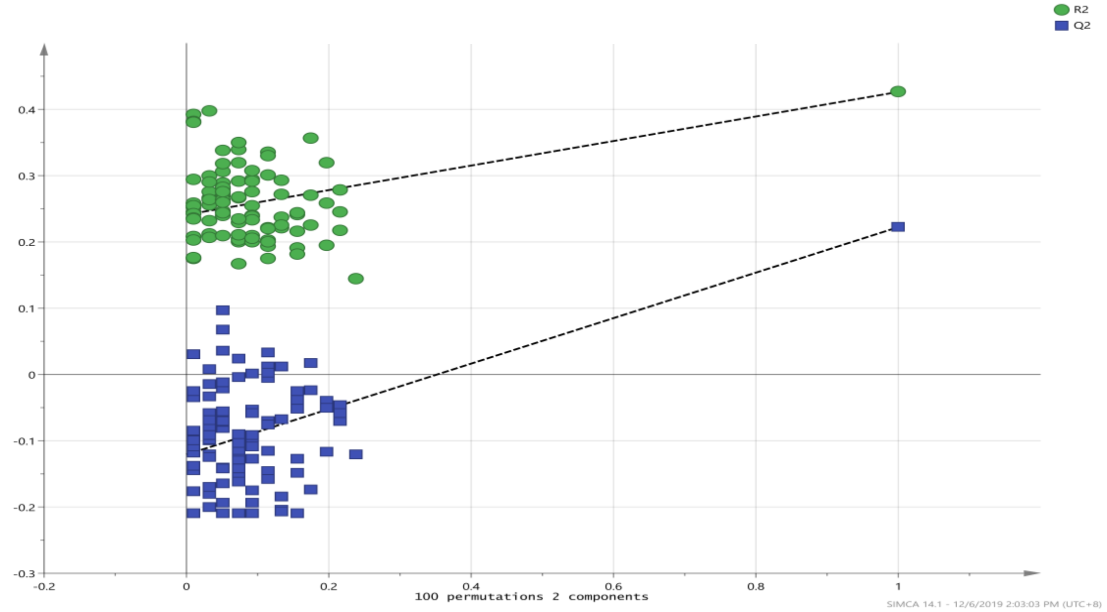

Figure S3. Permutation test for validation of PLS-DA model for (a) PEW groups R^2Y (0.0, 0.25), Q^2Y (0.0, -0.11) and (b) NPEW groups R^2Y (0.0, 0.24), Q^2Y (0.0, -0.12)

Note: Permutation test was performed to validate the supervised PLS-DA model and checked for an overfitting of the model. A total of 100 permutations were performed, and the resulting R^2 and Q^2 values were plotted with ● : R^2 and ■ : Q^2. The dash line represents the regression line for each value. The vertical axis gives the R^2Y and Q^2Y-values of each model while the horizontal axis represents the correlation coefficient between the ‘real’ Y and the permuted Y.

Table S1. ANOVA for cross validated residuals (CV ANOVA) of PLS-DA model discriminating PEW and NPEW groups

M4(PLS-DA)	SS	DF	MS	F	p	SD
**Total corrected**	96	96	1			1
**Regression**	21.15	4	5.29	6.50	<0.001	2.30
**Residual**	74.855	92	0.81			0.90

Abbreviations: SS: the sum of squares, DF: degree of freedom, MS: the corresponding mean squares, SD: standard deviations

Notes: Total corrected: SS of the Y of the training set corrected for the mean. Regression: fraction of total corrected SS accounted for by the PLS, here estimated by CV. Residual: difference between total corrected and regression SS, that is, the fraction of total corrected unaccounted for by the PLS model. The corresponding mean squares (MS), or variances are obtained by dividing each SS by the respective DF. The F-test, based on the ratio MS regression/MS residual then formally assesses the significance of the model. The p-value indicates the probability level for a model with this F-value being the result of just chance. CV-ANOVA is performed to calculate the p-value that estimates the significance of PLS-DA models with $p < 0.05$ is considered as significant model.

Table S2. Comparison on the mean concentration of discriminating metabolites identified from plasma samples of PEW and non-PEW HD patients

Metabolites	Spectra regions (ppm)	Mean PEW	Mean NPEW
**(A)**			
**1,3-Dihydroxyacetone**	4.42	0.042 ± 0.005	0.038 ± 0.005
**1,6-Anhydro-β-D-glucose**	5.46	0.121 ± 0.012	0.111 ± 0.013
**3-Hydroxy-3-methylglutarate**	2.42	0.054 ± 0.002	0.053 ± 0.001
**3-Hydroxybutyrate**	1.18	0.051 ± 0.008	0.024 ± 0.002
**Acetate**	1.9	0.188 ± 0.006	0.172 ± 0.005
**Arabinose**	5.3	0.228 ± 0.020	0.222 ± 0.034
**Ascorbate**	4.5	0.164 ± 0.012	0.157 ± 0.019
**Galactarate**	4.26	0.052 ± 0.005	0.049 ± 0.006
**Hydroxyacetone**	4.38	0.046 ± 0.002	0.045 ± 0.002
**Imidazole**	8.26	7.855 ± 0.126	7.585 ± 0.119
**Lactose**	4.46	0.138 ± 0.012	0.127 ± 0.014
**Lactulose**	4.34, 4.54	0.108 ± 0.008	0.104 ± 0.012
**Maltose**	5.38	0.174 ± 0.014	0.157 ± 0.022
**Mannose**	5.18	0.180 ± 0.020	0.153 ± 0.0180
**Ribose**	5.26	0.523 ± 0.035	0.459 ± 0.038
**S-Sulfocysteine**	4.18	0.159 ± 0.017	0.140 ± 0.014
**Sucrose**	4.22, 5.42	0.144 ± 0.117	0.111 ± 0.010
**Tartrate**	4.3	0.202 ± 0.027	0.182 ±0.032
**(B)**			
**Carnitine**	3.22	0.045 ± 0.001	0.054 ± 0.003
**Creatinine**	3.02, 4.06	0.270 ± 0.008	0.331 ± 0.010
**Galactose**	4.58	0.175 ± 0.016	0.173 ± 0.022
**Glucose**	3.38, 3.42 3.46, 3.5, 3.7, 3.74, 3.82, 3.86, 3.9, 4.62. 5.22	1.725 ± 0.058	1.999 ± 0.133
**Glycerol**	3.66	0.209 ± 0.008	0.213 ± 0.009
**Glycine**	3.54	0.191 ± 0.007	0.220 ± 0.014
**Guanidoacetate**	3.78	0.163 ± 0.005	0.178 ± 0.009
**Maltose**	4.66	0.143 ± 0.0124	0.138 ± 0.016
**Mandelate**	4.98	0.023 ± 0.001	0.022 ± 0.002
**Mannose**	4.9	0.168 ± 0.016	0.158 ± 0.185
**Methanol**	3.34	0.143 ± 0.047	0.156 ± 0.036
**Ribose**	4.94	0.488 ± 0.033	0.467 ± 0.410
**Trimethylamine N-oxide**	3.26	0.089 ± 0.003	0.101 ± 0.006
**π-Methylhistidine**	7.14	0.048 ± 0.003	0.052 ± 0.004

Notes: Plasma metabolites that discriminate PEW from NPEW group (a) and metabolites that discriminate NPEW from PEW group (b). The metabolites were quantified using the Chenomx 8.3 NMR Suite database. The values are arbitrary and expressed as mean ± SD.

Table S3. Analysis of Covariance (ANCOVA) between selected independent variables on significant metabolites with controlled covariates

Variables	Independent variable	df	F-test	*p*-value
3 Hydroxybutyrate	Group	1	10.251	0.002
	Gender	1	1.493	ns
	Group + Gender	1	1.537	ns
Acetate	Group	1	4.384	0.039
	Gender	1	0.365	ns
	Group + Gender	1	0.992	ns
Arabinose	Group	1	0.041	ns
	Gender	1	1.275	ns
	Group + Gender	1	0.307	ns
Maltose	Group	1	0.154	ns
	Gender	1	1.919	ns
	Group + Gender	1	0.566	ns
Ribose	Group	1	0.504	ns
	Gender	1	0.780	ns
	Group + Gender	1	0.122	ns
Sucrose	Group	1	3.333	ns
	Gender	1	0.462	ns
	Group + Gender	1	0.000	ns
Tartrate	Group	1	0.000	ns
	Gender	1	0.486	ns
	Group + Gender	1	0.504	ns
Creatinine	Group	1	28.518	<0.001
	Gender	1	10.516	ns
	Group + Gender	1	0.008	ns

Abbreviations: ns- not significant

Notes: This table shows the difference between groups and/or interaction effect between selected independent variables in term of significant metabolites after statistically control for covariates (age and dialysis vintage). *p*-values are derived using ANCOVA with Bonferroni correction, tested for adjusted means with $p < 0.05$ is considered statistically significant.
